# Application of Freire’s adult education model in modifying the psychological constructs of health belief model in self-medication behaviors of older adults: a randomized controlled trial

**DOI:** 10.1186/s12889-020-09425-7

**Published:** 2020-09-04

**Authors:** Kasra Gharouni, Arash Ardalan, Marzieh Araban, Farzad Ebrahimzadeh, Katayon Bakhtiar, Mohammad Almasian, Fatemeh Bastami

**Affiliations:** 1 School of Dentistry, Shiraz University of Medical Sciences, Shiraz, Iran; 2 Department of Cancer Registry, Providence Saint Joseph Medical Center, 501 S Buena Vista St, Burbank, CA 91505 USA; 3 Department of Health Education and Promotion, Public Health School, Ahvaz Jundishapur University of Medical Sciences, Ahvaz, Iran; 4 Department of Biostatistics and Epidemiology, Faculty of Health and Nutrition, Lorestan University of Medical Sciences, Khorramabad, Iran; 5 Department of Public Health, School of Health and Nutrition, Lorestan University of Medical Sciences, Khorramabad, Iran; 6 School of Medicine, Lorestan University of Medical Sciences, Khorramabad, Iran

**Keywords:** Elderly, Freire’s adult education model, Health belief model, Older adults, Self-medication

## Abstract

**Background:**

Self-medication by older adults has been always a public health concern. The present study aimed to modify the psychological constructs of Health Belief Model (HBM) in relation to self-medication behaviors using Freire’s Adult Education Model (FAEM) among older adults in Khorramabad, Iran, from 2017 to 2018.

**Methods:**

The mean age of the older adults was 66.28 ± 7.18 years. This was a randomized controlled trial study conducted on 132 individuals older than 60 who were referred from different health care centers. The participants were selected using multistage sampling method and randomly divided into two groups of intervention and control. The data collection instruments included a questionnaire which was designed based on both HBM and self-medication behaviors questionnaire. The phase of adult education model (AEM) was used to modify the psychological constructs of HBM and self-medication behaviors. Data were analyzed using SPSS software version 20 with a significant level of 0.05. Descriptive statistical tests, chi-squared test, paired t-test, independent t-test, and univariate modeling were employed for the purpose of analyzing data.

**Results:**

There was no significant difference between groups in terms of self-medication. Unawareness of the effects of medicine were the most important reason for self-medication (*p* = 0.50). The two groups were not significantly different in terms of knowledge, HBM constructs, and self-medication behaviors (*p* > 0.05). However, they came up to be considerably different for the above variables after the intervention was implemented (*p* <  0.05). When the findings were adjusted for the effects of confounding variables, there were significant differences between the two groups in almost all constructs of HBM and their behaviors (*p* <  0.05). However, the perceived barrier modality of HBM did not reach to a significant level of difference between two groups.

**Conclusion:**

The educational intervention, which was based on Freire’s AEM, had positive effects on the constructs of HBM and consequently on self-medication behaviors. The psychological constructs of HBM were affected at the phases of listening to problems. Self-medication was tempered at the action-reflection phase with shared creation and evaluation of the action plan geared toward the achievement of the behavioral objectives. The results might be of importance to healthcare professionals involved in care of older patients.

**Trial registration:**

Current Controlled Trials **IRCT2013091814512N2**. Registered on January 2 - prospectively registered, the trial was registered in the Iranian Clinical Trials Registry http://www.irct.ir.

## Background

Self-medication is defined as taking the commercially available medications. This is the most common form of self-care among people. Methods of getting hold of medications may include access to medicine without a prescription, using previously prescribed medications for similar cases, distributing prescribed medicine among family members and friends, as well as using leftover medicine [1, 2]. In addition, refusing to comply with prescriber’s instructions by taking relatively high doses of a medicine or for a long period of time, or even declining to complete the prescribed course of treatment can be considered under the same concept [3, 4]. Chronic diseases that mostly affect older people lead to pain, disability, decline in quality of life, and increased need for medications. Additionally, aging by itself is associated with increased side effects of medications because body is not able to metabolize them as well as before [5]. Likewise, several studies suggest that the costs of treatment rise as people get older [3, 6]. Previous studies show self-medication can cause even more harm than good among older adults; therefore, it would be fundamental to identify these behaviors and try to modify them so that older adults can have a long and healthy life [7].

According to a systematic review, self-medication behavior has been increasing in rates in the Middle East [8]. Arbitrary drug use rates in Iran are about three times of the global average. As such, Iran is among the first 20 countries in the world where self-medication is extremely common, and has ranked second only after China [9]. Investigations indicate that each Iranian national averagely takes averagely 339 tons of active substances annually, which is two to four times of world’s standard rate [10]. Although in most of the developing countries, there is access to medical treatment, WHO reports more than 50% of patients do not choose to go to a healthcare provider even it is necessary [11]. It is estimated that 83.3% of Iranians are currently using drugs arbitrarily [12]. In Iran, due to the pharmaceutical culture^1^ of the community and the relatively high prevalence of chronic diseases, older adults tend to use even greater amount of medications irrationally [3]. Older adults metabolize substances more slowly compared to young people. This may cause accumulation of medications in the body and therefore relatively high chance of over dosage of medicine or due toxicities [12]. This explains the challenges of arbitrary use of medicine among older adults of developing countries specially when hazardous medications are available over the counter and each older adult has multiple medications in his or her pocket [13]. The Elderly Health Program, which is a part of primary healthcare in the Iranian health system, does not currently include an educational intervention strategy to reduce arbitrary drug use [14].

Researchers have used models to identify reasons for behavioral changes related medication use. The Health Belief Model (HBM) is considered a comprehensive and effective program which helps achieve this goal. The main components of this model are based on psychological and behavioral theories. This model basically intends to identify and understand factors affecting behaviors as well as the way they may work. This model also offers ways to influence these factors under different circumstances [15]. Based on this model, if an individual is to change her or his behavior, she or he first has to be susceptible to a phenomenon, such as self-medication (perceived susceptibility); and understand the depth of related adverse effects on her or his life (perceived severity). Subsequently, she or he should admit the benefits of behavioral changes, and stop using medications on her or his own (perceived benefits). Likewise, she or he should be able to overcome existing barriers to taking action, such as costs (perceived barriers). The fifth structure of the Health Belief Model is called cue to action guides, which include accelerating forces that trigger one’s need for self-medication. Such cue guides can be internal such as understanding a physical state, or external like interpersonal interactions or media communication [16]. The efficacy of HBM in preventive health behavior has been confirmed in several studies [17–19].

Discussion and dialogs are among the cornerstones of changes in psychological behaviors [20]. Freire’s Adult Education Model (FAEM) is suggested to modify the psychological constructs of HBM. The essence of Freire’s model is dialog or skill acquisition through mutual communication. Freire’s model involves a three-phase process. In the first phase called Listening, the problem is set forth. In this phase, the various aspects of the issue are discussed with equal participation of all individuals. In the second phase called Reflection, emotional and social responses of individuals to the problem are emphasized. The last phase called Action-Reflection involves the joint creation of an operational plan for behavioral changes and its joint assessment [15, 21]. So far, several studies have been done to eradicate the habit of self-medication [22, 23] but only few have been conducted to evaluate experience-based education to eliminate self-medication behavior among older people. The objective of the present study was to modify knowledge, psychological constructs of HBM, and self-medication behaviors using adult education model among older adults residing in Khorramabad, Iran, from February 2017 to April 2018.

## Methods

### Study design

This was a randomized controlled trial registered at the Iranian clinical trials system under the code of 2,013,091,814,512 N2. The research was approved by the Ethics Committee of the Lorestan University of Medical Sciences, Khorramabad, Iran.

### Participants

Participants were recruited from ten different health centers in Khorramabad, Iran, from February 2017 to April 2018. Out of five postal districts in this town, health centers were randomly selected, so that individuals with different socioeconomic statuses were included in the study. The inclusion criteria consisted of being older than 60 years [24]^2^ residing in Khorramabad, Iran, being mentally healthy according to the Iranian health records, being able to perform daily activities independently, and meeting the criteria for self-medication behaviors. Criteria for self-medication behaviors included use of at least a non-prescribed medication for a chronic or acute illness, non-compliance with physician’s orders with respect to the prescribed medications such as raising or lowering medicine dosage, taking medicine for longer or shorter than recommended period of time, refusing to complete the course of treatment, and failing to take the medication on a timely manner. The exclusion criteria included suffering from neurological defects, emigration, failing to participate in more than one training session, or having psychological health deficits.

### Sampling and randomization

The multistage non-probability quota method was used for which the area of Khorramabad was divided into 3 strata of north, south and center. In each geographical stratum, there were some urban health centers. From the health centers of each stratum, two were randomly selected by systematic random sampling. A total of six health centers were selected out of the Khorramabad health centers. Finally, in each selected center, sampling was performed using the non-probability consecutive sampling method until the quota of each center was filled.

Patients were assigned to the intervention (*n* = 66) and control (n = 66) groups using permuted block randomization with a block size of 2 and an allocation ratio of 1:1. By use of random number table for each selected healthcare centers, a random sequence of “intervention” and “control” was generated. A column is randomly selected from the table of random numbers. The numbers are read from top to bottom. For numbers between 0 and 4, the words were sequenced as of “intervention” and then “control”. While, for the numbers between 5 and 9, the words were sequenced by “intervention” was followed by “control”. Finally, tables were installed in health centers, and the sampler selected intervention or control as the first experimental group for the first qualified person. Obviously, the next word should be used based on the random sequence method [25]. The primary sample size in each group (intervention and control) was calculated as 63 individuals using the following formula:

$$ n=\frac{{\left({z}_{1-\raisebox{1ex}{$\alpha $}\!\left/ \!\raisebox{-1ex}{$2$}\right.}+{z}_{1-\beta}\right)}^2\left(2{s}^2\right)}{d^2} $$

z_1-α/2_: 97.5th percentile of the standard normal distribution corresponding to type I error probability of 0.05 = 1.96.

z_1-β_: 80th percentile of the standard normal distribution corresponding to test power of 0.90 = 0.84.

s: an estimate of the standard deviation of total performance score change in both groups = 2.00

$$ \left({\sigma}_{change}=\sqrt{\sigma_{before}^2+{\sigma}_{after}^2-2{\rho \sigma}_{before}{\sigma}_{after}}\simeq \sqrt{2^2+{2}^2-2\times 0.5\times 2\times 2}\simeq 2.00\right) $$

d: the minimum mean difference of total performance score change between the two groups, which is of great importance in the opinion of the researcher = 1. Ultimately, considering the probability of 5% for loss of samples, the final sample size in each group was estimated to be 66.

Researchers contacted the selected older adults through the contact information available in their records and asked them to appear at the assigned health centers for face-to-face interviews. After the interviews, 13 people were excluded from the study due to lack of self-treatment behavior and other entry criteria. Criteria for self-medication behaviors included use of at least a non-prescribed medication for a chronic or acute illness, non-compliance with physicians’ orders with respect to the prescribed medications such as raising or lowering medicine dosage, taking medicines for longer or shorter than the recommended period of time, refusing to complete the course of treatment, and failing to take medications in a timely manner. Four of them were reluctant to participate in the study. Three of them were also unable to perform their activities independently due to neurological defects or mental disorders such as dementia. Therefore, they were unable to take care of themselves so that we could not investigate the arbitrary use of medicine among them. Therefore, the collected data might not have been reliable if they were included.

After informed consent was obtained by a healthcare worker who was not involved in the recruitment of participants, the older adults were randomized into control and intervention groups by permuted block randomization and a table of random numbers [26]. The older adults who met the criteria of self-medication behavior filled out the pre-test questionnaires. The corresponding author and research assistants collected the data. The research assistants were graduate students majoring in public health. In February and March 2017, participants were contacted for face-to-face interviews and to assess their eligibility to participate. In April, May and June 2017, the questionnaires were completed by both intervention and control groups. In July and August 2017, training sessions were held up. The participants in the intervention group received an educational intervention about the regular use of medications in addition to the routine integrated health care services for older adults. Six months after the intervention was conducted, a researcher who was blinded to group allocation administered the follow-up questionnaire. The post-test questionnaire was completed in March and April 2017.

### Intervention

The intervention was carried out according to FAEM and included six 45-min educational sessions. The number of training sessions was determined based on the method proposed by Clark et al., which was performed on older adult populations [27]. The adult learning process was implemented in the three phases listed below.

### Listening phase

The facilitator raised the issue of self-medication by playing a video about older adult individuals suffering from liver and kidney complications due to self-medication. Brainstorming and subsequently a group discussion were held among the older adults so that they got a chance to share their experiences regarding self-medication behaviors. This way, perceived susceptibility was applied. The older adults were advised about the need for referring to physicians in the event of chronic or acute illnesses, and the necessity of refraining from obtaining medications from pharmacies without a prescription. They were also alerted against the use of herbal and traditional medicines without a physician’s prescription. Then, perceived severity was persuaded by presenting statistical information and a case study that emphasized the negative consequences of self-medication. The older adults talked about the various complications associated with self-medication such as prolongation of the disease, increased costs of treating illnesses as a result of self-medication, psychological and social burden of losing health such as reduced overall life satisfaction, and limited communication with friends and family.

### Reflection phase

In each group, the older adults discussed potential barriers by brainstorming. They role-played about the barriers under discussion followed by a discussion about the solution to the obstacles that were role-played. In this phase, some beliefs were brought up, including the idea that health is so important that some time and money should be put aside for visiting doctors, the treatment offered by doctors should be trusted, and that even if they experience severe pain and do not have access to a physician, they should not self-medicate. To modify the structure of perceived benefits, a series of instructions for taking medications such as proper dosage, timing, and course of treatment were discussed with individuals who had chronic illnesses. A discussion was subsequently held on the benefits of refraining from self-medication, including medication safety and receiving quality treatment through medications prescribed by a physician. At the end of this phase, the older adults expressed their emotional and social concerns about self-medication behaviors. Eventually, the older adult individuals discussed how they could resolve this issue.

### Action-reflection phase

The behavioral objectives of modifying self-medication behavior were set at this stage. According to these objectives, older adults should refrain from taking medications without the prescription of a physician. Likewise, older adults should abstain from prolonging or shortening the duration of treatment and complete the course of treatment. Older adult individuals should also avoid increasing or decreasing the medication dosage. The older adults were given recall cards that could be put in the most visible places, such as on refrigerators in order to remind them to take their medications on time. They read consumer medication information leaflets before taking medications and disposed expired medications. They also created a virtual group with their peers, family members, and health caregivers to monitor their regular use of medicine while sharing their experiences and educational materials. They also increased the number of authoritative sources such as materials by the Ministry of Health and from health caregivers by which they acquired knowledge on the correct use of medications. Thereby, the construct of external cues to action was modified. Additionally, they paid proper attention to their achieved general health because of regular use of medications as an internal cue to action.

### Outcome measures

The primary outcome was increased knowledge and improved attitude towards self-medication, measured by a questionnaire assessing the participants’ knowledge and constructs of HBM at baseline and 6 months following the intervention. The HBM scale has been used in a previous descriptive study of self-medication in older adults [19].

Part one describes older adults’ knowledge of self-treatment. A score of 1 indicates a correct answer while a score of 0 is considered a wrong one. Part II includes questions that assess the health belief model constructs. Five items about perceived sensitivity measured a person’s belief if they were susceptible to self-medication (Example: I may self-medicate when I am ill). Five items about perceived severity assessed one’s belief about the harmful effects of self-treatment on life (Example: I believe self-therapy may in some cases lead to death). Five items concerning perceived benefits measured one’s belief in the usefulness of the correct use of medications (Example: I believe that with the proper use of medications the duration of illness is shorter). Four items on perceived barriers assessed one’s perceived ability to cope with challenges and barriers to proper use of medications (Example: Due to the high cost of a physician visit, I cannot see a physician during illness). This questionnaire was rated on a 5-point Likert scale from strongly agree to strongly disagree. “I strongly agree” scores 4, “agree” scores 3, “no idea” scores 2, “disagree” scores 1, and “strongly disagree” scores 0. Then, the obtained score is expressed as a percentage. The total score ranged from 0 to 100.

The fifth construct of Health Belief Model is called cues to action, which guide one’s need or actions with regard to self-medication. Internal and external cues to action have been accounted in the form of frequency distributions. The HBM questionnaire has been used in studies by Karimi and Sharifirad performed on older adults so that the validity and reliability of the questionnaire has been confirmed [28, 29].

Section 3 includes reasons for self-treatment as assessed by a 14-item checklist. This questionnaire was answered by yes or no, and reported through frequency distribution. The secondary outcome included a reduction in self-medication behaviors which was assessed at baseline and six months after the intervention. A questionnaire was used including a series of dimensions for the purpose of considering behavioral objectives, and measuring self-medication behaviors. The dimensions included refraining from using medications without physicians’ prescriptions, refraining from prolonging or shortening the duration of treatment, refraining from increasing or decreasing dose and the regular use of medications according to the time assigned for each. Each dimension was evaluated with two questions about chronic and acute illnesses. The options to each question measured the frequency of the said behaviors from Never to More than four times.

### Validity

The Health Belief Model provided a conceptual framework for designing the questionnaire. A set of items that measure health belief model constructs on self-medication were created. This model accurately illustrates the relationship between health beliefs and behaviors. This model is specifically tailored for prevention-based interventions as well as interventions that make short-term changes [30]. The validity of this questionnaire was evaluated by the following method [31, 32].

### Face validity

Both qualitative and quantitative methods were used for face validity. For the purpose of qualitative approach, 20 participants were asked to evaluate each item for imprecision and complexity. In general, there were no problems in reading and understanding the items by participants. The quantitative face validity was assessed by impact scoring. The impact score for each item was calculated by multiplying the importance of an item by its frequency. The impact scores of greater than 1.5 were considered suitable [33].

### Content validity

An expert panel including experts such as a health education expert, a statistician, an epidemiologist, a gerontologist, a pharmacologist and a general practitioner examined the content validity. The expert panel was asked to examine each question based on the three-part spectrum “necessary”, “useful but not necessary” and “not necessary”. The results were used to calculate the content validity ratio (CVR) using the following formula:

The numbers of specialists who have chosen the necessary option/half of all evaluators.

**----The Formula----**

$$ CVR=\frac{n_E-\frac{N}{2}}{\frac{N}{2}} $$

Based on Lawshe’s table, the expressions for which the CVR value was higher than 62.0 were retained for later stages [34]. Next, ten experts were asked to comment on three characteristics of the questionnaire, including relevancy, simplicity and clarity in a 4-part Likert scale. For example, for the sake of clearance grading, they scored like “is not clear=1 is relatively clear=2, is clear=3, and is quite clear=4”. Then, content validity index (CVI) for each item was calculated by dividing the number of experts who agreed with grades 3 and 4 by the total number of specialists. Finally, items with a CVI value greater than 0.79 were reserved [31].

### Reliability

The internal consistency of the instrument was assessed using Cronbach’s alpha coefficient [35]. Alpha values equal or greater than 0.70 were considered as satisfactory.

### Statistical analysis

Stratification variables have been presented as frequencies and percentages. They were compared by the chi-square test. Continuous variables have been presented as means and standard deviations and tested by paired t-test after evaluating their normality. In order to reduce the effects of confounding variables, two different ANOVA models were used. They include an ANOVA model which was only adjusted for basic variables. The second ANOVA model was adjusted for basic as well as demographic variables. Statistical analyses were performed using SPSS software version 20.0 (IBM) at the significance level of 0.05.

### Ethical considerations

This research project has been registered at the Research Committee of Lorestan University of Medical Sciences with the registration number 2040. It was reviewed and approved by the Ethics Committee of the Lorestan University of Medical Sciences. All the participants took part in this study voluntarily. Participants signed a consent form before participation. In order to observe the principles of ethics in research, the educational materials were also made available to the control group at the end of the study.

## Results

The Kolmogorov-Smirnov test showed the quantitative variables followed a normal distribution (*P* > 0.05). In terms of demographic characteristics, there was no significant difference between the intervention and control groups (Table 1). There was no significant difference between the two groups in terms of self-medication causes. Lack of adequate knowledge about the harmful effects of medicine was the most important cause of self-medication in both groups (Table 2). The mean scores of knowledge, psychological constructs of health belief model and self-care behaviors improved significantly after intervention in the related group (Table 3). Table 4 shows the results of univariate modeling in two models. The univariate model showed that there were no significant differences between the intervention and control groups after eliminating the effects of pre-intervention measurements, except for the cases of behaviors (*P* > 0.001) and perceived benefits (*P* = 0.002). Model 2 shows intervention measurement values as well as demographic variables following adjustment.

**Table 1:** The relative frequency distribution of the participants based on age, gender, educational attainment level, marital status, income, and insurance status in the control and intervention groups

Demographic Characteristics	Number(132)		***P***-Value*
	Control Number (Percent)	Intervention Number (Percent)	
**Gender**			
Male	33 (50%)	34 (51.5%)	0.50
Female	33 (50%)	32 (48.5%)	
**Age** (years)			
60–69	56 (84.8%)	47 (71.2%)	0.092
70 and older	10 (15.2%)	19 (28.8%)	
**Education**			
Sub-secondary	57 (86.4%)	59 (89.4%)	0.66
Secondary	6 (9.1%)	5 (7.6%)	
University	3 (4.5%)	2 (3%)	
**Marital Status**			
Single, Widow, Widower	12 (18.2%)	19 (28.8%)	0.10
Married	54 (81.8%)	47 (71.2%)	
**Income**			
Low	20 (30.3%)	31 (47%)	0.14
Middle	16 (24.2%)	12 (18.2%)	
Good and High	30 (45.5)	23 (34.9%)	
**Insurance Status**			
Insured	46 (69.7%)	43 (65.2%)	0.35
Without Insurance	20((30.3%)	23 (34.8%)	
**Occupation**			
Employed	12 (18.2%)	10 (15.2%)	0.82
Housewife	33 (50%)	32 (48.5%)	
Retired	21 (31.8%)	24 (36.4%)	

*derived from chi-square

**Table 2:** The frequency distribution of reasons for self-medication

Reasons for Self-medication	Intervention	Control	***p***-Value*
	Number (%)	Number (%)	
The Insistence of Others	21 (31.8%)	23 (34.8%)	0.43
Lack of Access to a Physician	18 (27.3%)	16 (24.2%)	0.42
High Costs of Visiting a Doctor	24 (36.4%)	21 (31.8%)	0.36
Considering the Disease as not Serious	23 (34.8%)	27 (40.9%)	0.30
Lack of Enough Time for Referring to a Physician or Hospital	14 (21.2%)	23 (34.8%)	0.06
Previous Experience with the Disease	38 (57.6%)	40 (60.6%)	0.43
Availability of Medicines (at home, from friends and acquaintances, etc.)	34 (51.5%)	38 (57.6%)	0.30
Being Able to Easily Obtain Drugs from Pharmacies without the Need for Prescriptions	18 (27.3%)	27 (40.9%)	0.07
Not Having Health Insurance	9 (13.6%)	8 (12.1%)	0.50
Not Trusting Physicians	8 (12.1%)	10 (15.2%)	0.40
Not Knowing Enough About the Effects of Drugs	40 (60.6%)	39 (59.1%)	0.50
The Belief that Drugs do not Have Side Effects	27 (40.9%)	18 (27.3%)	0.07
N	132 (100%)		

*Derived from chi-square

**Table 3:** The comparison of the mean scores of awareness, perceived susceptibility, perceived severity, perceived benefits, perceived barriers, and self-medication behaviors in the intervention and control groups before and after the intervention

Variables	Groups	Before Intervention	After Intervention	p-value^b^	Mean Difference ± SD	95% confidence interval for Difference	
		Mean ± SD	Mean ± SD			Lower	Upper
Awareness	Intervention Control	48.63 ± 18.21 44.45 ± 19.86	82.87 ± 12.24 45.3 ± 19.70	< 0.001 0.44	−34.25 ± 19.77 −6.66 ± 10.86	−39.10 −9.33	−29.38 − 0.99
	p-value^a^	0.85	< 0.001				
Perceived Susceptibility	Intervention Control	47.65 ± 15.49 48.10 ± 17.02	78.79 ± 17.58 50.12 ± 15.71	< 0.001 0.59	−31.14 ± 24.65 −2.05 ± 31.33	−37.19 −9.75	− 2508 5.66
	p-value^a^	0.87	< 0.001				
Perceived Severity	Intervention Control	49.53 ± 16.08 45.46 ± 13.38	62.22 ± 14.05 44.51 ± 11.47	< 0.001 0.3	−12.70 ± 20.98 1.14 ± 4.09	−17.85 0.13	−7.54 2.14
	p-value^a^	0.13	< 0.001				
Perceived Benefits	Intervention Control	53.10 ± 19.44 55.75 ± 6.39	75.75 ± 11.24 56.89 ± 5.99	< 0.001 0.2	−22.65 ± 17.83 −1.14 ± 2.89	−27.03 − 1.85	−18.26 −0.43
	p-value^a^	0.29	< 0.001				
Perceived Barriers	Intervention Control	58.10 ± 17.66 50.60 ± 18.19	53.33 ± 18.48 50.83 ± 16.88	0.04 0.81	4.78 ± 19.18 −0.23 ± 7.77	0.06 −2.13	9.49 1.67
	p-value^a^	0.01	0.41				
Self-Medication Behavior	Intervention Control	45.74 ± 11.98 48.48 ± 17.59	15.53 ± 5.82 45.92 ± 15.44	< 0.001 0.20	30.21 ± 12.50 2.56 ± 4.35	27.14 1.49	33.28 3.63
	p-value^a^	0.29	< 0.001				

^a^ Significant, Independent -samples t-test

^b^ Significant, Paired-samples t-test

**Table 4:** The results of modeling the effects of the educational intervention on awareness, the constructs of HBM, and behavior, taking the effect of time into account

Variable	The intervention group		The control group		p-Value in Model 1	p-Value in Mode 2
	Before	After	Before	After		
	Mean ± SD	Mean ± SD	Mean ± SD	Mean ± SD		
Awareness	48.63 ± 18.21	82.87 ± 12.24	44.45 ± 19.86	45.30 ± 19.70	0.27	< 0.001**
Perceived Susceptibility	47.65 ± 15.49	78.79 ± 17.58	48.10 ± 17.02	50.15 ± 15.71	0.4	0.02*
Perceived Severity	49.53 ± 16.08	62.22 ± 14.05	45.46 ± 13.38	44.51 ± 11.47	0.5	< 0.001**
Perceived Benefits	53.10 ± 19.44	75.75 ± 11.24	55.75 ± 6.39	56.89 ± 5.99	0.002**	0.003**
Perceived Barriers	58.10 ± 17.66	53.33 ± 18.48	50.60 ± 18.19	50.83 ± 16.88	0.14	0.03*
Behavior	45.74 ± 11.98	15.53 ± 5.82	48.48 ± 17.59	45.92 ± 15.44	< 0.001**	< 0.001**

Model 1: The model adjusted for the pre-intervention measurements

Model 2: The model adjusted for the pre-intervention measurements, gender, age, educational level, occupation, income, and insurance status

Among participants, 56 participants (42.4%) mentioned recall cards, 53 (40.2%) of elderlies stated families and acquaintances, 14 (10.6%) declared physicians, and 6 (6.8%) pointed out television as the most important external cues to action. This adds up to 129 individuals, while three participants refused to talk about it. Likewise, 94 persons (71.2%) acknowledged fear of being affected by the adverse effects of self-medication, 22 participants (16.7%) confirmed not believing in self-medication, and 16 (12.1%) individuals affirmed general good health as the most important internal cues to action which persuaded them to undertake preventive behaviors. This added up to 132 individuals.

## Discussion

The present study aimed to modify knowledge, psychological constructs of HBM, and self-medication behaviors using adult education model. The results showed there was a significant difference between intervention and control groups after the intervention, when the effects of confounders such as demographic variables, knowledge, behaviors, and the constructs of HBM were controlled. According to the results of the final multivariate model, the educational intervention had a significant impact on self-medication behaviors. The application of HBM in combination with Freire’s adult education model (FAEM) enhanced its effectiveness in older adults. According to Peyman et al., educational intervention in the framework of Freire’s education model created favorable changes in attitudes and eating disorders of students [36]. The main point in Freire’s model is the problem-posing technique. This approach is completely different from traditional educational methods. In Freire’s model, there are no pre-made solutions and individuals look for a solution by considering different aspects of problems [37].

According to a previous study, HBM affects behaviors through several factors. A study was designed using SMS wording based on HBM to improve adherence to treatment in low-resource settings. The study showed that behaviors would change by SMS wording which effectively touches on the themes of HBM [38]. Moreover, in another trial that was conducted to reduce self-medication behaviors among hypertensive older adults, a personal educational program designed to promote adherence to treatment through psychological constructs led to a change in intent; thus, improved behaviors [39]. The findings of the present study are consistent with two other studies by Shamsi and Movahedi [40, 41].

The intervention had three different phases. In the Listening Phase, the older adults encountered the subject of self-medication through strategies used in this phase which increased sensitivity and perceived severity toward the subject. It was observed that the majority of participants in the intervention group came to believe that they were susceptible to the adverse effects of self-medication after the intervention. This finding is consistent with the results of three different studies by Moghadam, Niksadat and Heydartabar [42–44]. As long as an older adult finds herself or himself more susceptible to diseases caused by self-medication, she is more likely to take precautionary measures. According to previous studies, perceived severity was a predictor of self-medication behaviors among older adults [19, 45]. In the present study, the perceived severity of older adults increased in terms of the extent of harm caused by the arbitrary use of medications. Likewise, in a study conducted on pregnant women, following an educational intervention, the participants were considerably more concerned about the side effects of self-medication during pregnancy, the consequent birth of an abnormal baby and the emergence of domestic problems [40].

At the end of the listening phase, significant increases were observed in the older adults’ knowledge of definition of self-medication, variables such as the adverse effects of medicine, organs susceptible to self-medication, correct method of keeping medications, and recognizing expired drugs. According to previous studies, mutual learning with feedback from other learners is more effective and enduring. In fact, good knowledge is a predictor of positive attitude, while a relationship was found between knowledge and performance of older adults [8]. This issue also emphasizes the need for retraining programs for physicians and pharmacists, so that physicians can put more effort into educating older adults about proper use of medications. In the present study, inadequate knowledge about the harmful effects of medications was the most important reason for self-medication in both intervention and control groups. In line with the current study, a systematic review showed that most patients do not have enough knowledge about active agents, as well as the methods of taking medications, and the side effects of medications. The majority of patients (60%) do not read consumer information leaflets of medicines enclosed within the drug packaging [8].

The second phase called Reflection was about finding an answer to the question of how older adults can explain the situation and take control of it. In this phase, the costs of and barriers to performing the behaviors and possible solutions to overcoming them were discussed by brainstorming. Thereby the constructs of perceived barriers and benefits were modified. As such, perceived barriers were found to be a predictor of self-medication behaviors among older adults. According to a meta-analytic study by Carpenter, it was generally suggested that the construct of perceived barriers in HBM is an important factor to prevent or avoid unhealthy behaviors [46]. The construct of perceived barriers refers to beliefs about the actual and feasible costs of refraining from arbitrary use of drugs, which declined after intervention in the intervention group.

Studies on the construct of perceived barriers have yielded inconsistent results [40, 41, 47]. This can be attributed to the variety of perceived barriers such as financial, physical, psychological, and social barriers, as well as the different effects of educational interventions on modifying these barriers [48]. According to univariate modeling, when the effects of demographic variables were taken into account, the impact of educational intervention on perceived barriers did not seem to be significant. On the other hand, the construct of perceived benefits increased the relative efficacy of known methods used by older adults to avoid self-medication after the intervention. Indeed, understanding the proper use of medicine can reduce their adverse effects and help quick recovery from certain diseases, which can be effective in improving perceived benefits. This finding was similar to that of the study by Shamsi which was conducted on pregnant women in Arak, Iran [40]. However, based on a study by Movahedi, no significant changes occurred following intervention [41]. This could have been due to long term adverse effects of unhealthy behaviors which were intangible for the students participating in the study.

In the Action-Reflection phase, behavioral goals were set to modify self-medication behaviors. After the educational intervention, self-medication behaviors decreased significantly in the intervention group compared to the control group. Proper medication use was the final result of the intervention in which the conceptual framework of adult education was used. According to previous studies, behavioral habits such as regular use of medications in accordance with physicians’ prescriptions influenced the development of these behaviors [39, 49]. Recall cards were the main cue for action for older adults on the correct use of medications. According to a study conducted to reduce car accidents, putting labels containing messages in front of drivers’ eyes was the best method of information delivery. In some studies, family members, friends and healthcare providers have been considered as role models and as the most important promoters of health. The participants also shared their experiences with friends, family members, and caregivers in virtual groups in order to help monitor their regular use of drugs. As a result, the participants acted intelligently in regard to medication use.

After discussing how the educational intervention was implemented in this study, it is necessary to talk about the types of diseases that older men and women mostly self-medicated for and the most frequently used medications. According to the study results, headaches and cold accounted for the highest rates of self-medication among both men and women. In addition, the use of antibiotics, cold medicines and painkillers had the highest frequency in both men and women. Previous studies also indicate colds and headaches are the most common diseases that are self-medicated in Iran. In addition, this study showed that analgesics, antibiotics and cold medicines are the most common drugs that are used for self-medication [12]. Likewise, a systematic review has shown painkillers and antibiotics are the main medicine that are used in self-medication in the Middle East. This systematic review, found 91% of Iranians with migraine headaches take acetaminophen and codeine without a doctor’s prescription. Yuz reported 32 to 42% of people of other counties in the Middle East take antibiotics with no prescription compared to 53% of Iranians [8]. The studied populations have limited knowledge about which infections require antibiotics and which antibiotics are safe enough to be taken with no prescription. For example, a study found that 67.1% of the general population believe cough and cold require antibiotics for treatment, and many people use antibiotics for viral infections as well. Likewise, 28.1% of the studied individuals used antibiotics instead of painkillers. In addition, many people were identified as being poorly compliant with antibiotics because they did not finish the treatment course, but took antibiotics for a couple of days only [50].

In the care defined for elderlies in the national system of registry in Iran called SIB, the strategy of educational intervention on the arbitrary use of drugs is not yet considered. Thus, it is suggested that educational intervention programs on the correct use of drugs be provided in comprehensive health service centers based on theories suitable for the elderly [14].

The first highlight of the conceptual framework of Freire’s adult education used in this study is to establish two-way communication between older adults. Since discussing the issue is the essence of this approach, it is also called collaborative research. The second highlight of the methodology of this conceptual framework was problem design. Older adult learners should search for solutions themselves and implement the solutions in an action plan.

### Limitations

This study had some limitations. The self-medication questionnaire was a self-report questionnaire. The researchers were limited to collecting required data by checklists and questionnaires, so the data were just reported by older adults; thus, the findings might have been affected by recall bias. However, according to previous research, self-reporting is considered a common method of evaluating performance in the behavioral sciences [19, 29, 51–53]. This study did not make a distinction between over-the-counter medications, e.g. pain-killers, medications prescribed for chronic diseases e.g. diabetes and those that require prescription but should only be taken in a short space of time e.g. antibiotics. Future studies are recommended among older adults considering these limitations. Given that the adult education approach has been used in a limited number of studies, the discussion of the results was limited in scope. Therefore, it was attempted to compare and discuss the results of closely related studies.

## Conclusion

More attention should be paid to the growing risk of drug abuse for illnesses such as cold and pain. Training physicians to prescribe the right medications is an approach that may encourage the appropriate use of antibiotics and painkillers. In addition, an appropriate policy must be implemented to limit the sale of over-the-counter drugs. The framework of FAEM was suitable for modifying the psychological constructs of HBM with regard to self-medication behaviors among older adults. The constructs of perceived susceptibility and perceived severity were modified by subjecting aspects of the issue of self-medication to discussion. The construct of perceived benefits and barriers were modified by discussing possible solutions and their benefits. Older adults can use medicines wisely by creating a joint action plan geared towards the behavioral objectives. It is recommended that health personnel are trained to apply the adult education framework in comprehensive health centers and nursing care centers so that health behaviors such as adherence to treatment and proper drug use may improve.

## Data Availability

The datasets used and/or analyzed during the current study are available from the corresponding author on reasonable request.

## Abbreviations

HBM: Health Belief Model
FAEM: Freire’s Adult Education Model

## Footnotes

1 Unfortunately, people can buy medicines from a pharmacy with no prescription in Iran. The pharmaceutical culture means the way most of the people think about taking medicine; whether it is prescribed by a physician or those are just collected from a pharmacy based on their own thoughts.

2 According to the World Health Organization(WHO), the age of onset for being an older adults is defined as 60.
